# LncRNA IGFL2-AS1 as a ceRNA Promotes HCT116 Cell Malignant Proliferation via the miR-433-3p/PAK4 Axis

**DOI:** 10.5152/tjg.2023.21998

**Published:** 2023-05-01

**Authors:** Shuang Liu, Xiangjie Wang, Jing Zhang, Yabin Liu, Binghui Li

**Affiliations:** 1 Department of General Surgery, The Fourth Hospital of Hebei Medical University, Shijiazhuang, Hebei, China; 2 Department of Neurology, The Fourth Hospital of Hebei Medical University, Shijiazhuang, Hebei, China

**Keywords:** ceRNA, colorectal cancer, incRNA IGFL2-AS1, malignant proliferation, miR-433-3p, PAK4

## Abstract

**Background:**

Colorectal cancer is a common gastrointestinal malignancy worldwide. Many studies have proved that long noncoding RNA alterations participate in colorectal cancer development. This study sought to probe the regulatory mechanism of lncRNA IGF-like family member 2 antisense RNA 1 (IGFL2-AS1) in colorectal cancer cell malignant proliferation.

**Methods:**

LncRNA IGFL2-AS1 expression in colorectal cancer and para-cancerous tissues and colorectal cancer cell lines was detected via quantitative real-time polymerase chain reaction. HCT116 cells were transfected with si-IGFL2-AS1, microRNA (miR)-433-3p inhibitor or p21 (RAC1)-activated kinase 4, PAK4 and IGFL2-AS1 overexpression vector, followed by assessment of cell proliferation and clone formation using 3-[4,5-dimethylthiazol-2-yl]-2,5 diphenyl tetrazolium bromide assay and colony formation assay. The subcellular localization of lncRNA IGFL2-AS1 was predicted and testified via the nuclear/cytosol fractionation assay. The downstream miRNA of lncRNA IGFL2-AS1 and downstream target of miRNA were predicted, and their binding relationships were testified using dual-luciferase assay and RNA immunoprediction experiment.

**Results:**

LncRNA IGFL2-AS1 was abundantly expressed in colorectal cancer tissues and cells. Silencing lncRNA IGFL2-AS1 discouraged HCT116 cell malignant proliferation, while lncRNA IGFL2-AS1 overexpression played an opposite role. LncRNA IGFL2-AS1 was mainly expressed in the cytoplasm of HCT116 cells. LncRNA IGFL2-AS1 bound to miR-433-3p and inhibited its expression. miR-433-3p targeted PAK4. Silencing lncRNA IGFL2-AS1 facilitated miR-433-3p to suppress PAK4 transcription. miR-433-3p inhibition or PAK4 overexpression partly reversed the inhibition of silencing lncRNA IGFL2-AS1 on HCT116 cell malignant proliferation.

**Conclusion:**

LncRNA IGFL2-AS1 was abundantly expressed in colorectal cancer tissues and cells, and comparatively bound to miR-433-3p to facilitate PAK4 transcription, thus promoting HCT116 cell malignant proliferation.

**Main Points**

- Long noncoding RNA insulin-like growth factor 2-like family member 2 antisense RNA 1 (LncRNA IGFL2-AS1) is highly expressed in colorectal cancer (CRC) tissues and cells.
- Silencing lncRNA IGFL2-AS1 inhibits HCT116 cell proliferation.
- LncRNA IGFL2-AS1 comparatively binds to miR-433-3p to upregulate p21 (RAC1)-activated kinase 4 (PAK4).
- Decreased miR-433-3p or elevated PAK4 reverses the role of silencing IGFL2-AS1.
- LncRNA IGFL2-AS1 promotes HCT116 cell proliferation via the miR-433-3p/PAK4.

## Introduction

Colorectal cancer (CRC) results from the uncontrolled proliferation of intestinal epithelial cells; in global cancer statistics, CRC ranks third by incidence and second by mortality, causing 10.0% of new cancer cases and 9.4% of oncological deaths worldwide.^1,2^ Age, genetic, and environmental factors are major risks for the pathogenesis of CRC.^3^ Surgical resection remains the cornerstone of CRC management, and adjuvant chemotherapy is conducive to eliminating micro-metastases.^4^ However, due to the high rate of metastasis and recurrence, the long-term survival rate of CRC patients has been little improved by existing treatment options.^5^ Therefore, the genetic landscape of CRC remains to be investigated to discover novel prognostic hallmarks and therapeutic targets for CRC.

Long noncoding RNAs (lncRNAs), transcripts longer than 200 nucleotides without protein-coding capacity, could function as microRNA (miRNA) (the small transcript with about 22 nucleotides in length) sponges, also known as competing endogenous RNAs (ceRNAs), to regulate the biogenesis of mRNAs.^6^ In the clinical setting of CRC, lncRNAs could serve as biomarkers for early diagnosis, prognostication, and targeted treatment.^5^ Long noncoding RNA insulin-like growth factor 2-like family member 2 antisense RNA 1 (IGFL2-AS1) is a newly emerging oncogene that is ectopically expressed in various cancer types, such as gastric cancer, tongue squamous cell carcinoma, and colon cancer.^7-9^ However, the expression pattern and role of lncRNA IGFL2-AS1 in CRC have not been researched before. In the current study, lncRNA IGFL2-AS1 was found to have an upregulation in CRC tissues and cells. Hence, we investigated the regulatory mechanism of lncRNA IGFL2-AS1 in CRC.

Long noncoding RNA IGFL2-AS1 was previously highlighted to be a ceRNA via binding to miRNA response elements to play a role in regulating its downstream targets.^8,10^ miR-433, especially miR-433-3p, derived from chromosome 14q32.31, has been regarded for its prognostic value in cancers, such as non-small cell lung cancer, bladder cancer, and colon cancer.^11-13^ Hard-done work of our peers has shed light that miR-433 overexpression suppresses CRC cell proliferation, migration, and invasion, and its reduced level is indicative of advanced tumor stage and early relapse of CRC patients.^14,15^ miR-433-3p was previously demonstrated to target p21 (RAC1)-activated kinase 4 (PAK4) in oral squamous cell carcinoma.^16^ p21 (RAC1)-activated kinase 4, an oncogenic component of the PAKs family, is overexpressed in cancers upon overcoming oncogene-induced senescence.^17^ In CRC, PAK4 promotes cancer development while its oncogenic function is often reversed by its target miRNAs.^18,19^ Nevertheless, the mechanism of the ceRNA network of lncRNA IGFL2-AS1/miR-433-3p/PAK4 in CRC has not been discussed before.

In light of the aforementioned data, we hypothesized that lncRNA IGFL2-AS1 regulates CRC cell malignant proliferation via manipulation of miR-433-3p and PAK4. Consequently, our study sought to evaluate the role of lncRNA IGFL2-AS1 as a therapeutic target in CRC and confer a novel theoretical basis for the suppression of CRC cell malignant proliferation.

## MATERIALS AND METHODS

### Clinical Sample Acquisition

Cancer tissues and para-cancerous tissues were collected from 63 CRC patients (37 males and 26 females) who were confirmed with diagnosis and received surgical treatment in the Fourth Hospital of Hebei Medical University from April 2018 to April 2020. All included patients were diagnostically confirmed by the Fourth Hospital of Hebei Medical University and did not receive any anti-cancer treatment before surgery or were diagnosed with other malignancies or immune diseases, etc. During surgery, the collected tissues were confirmed by at least 2 pathologists. Resected tissues were frozen in liquid nitrogen at −80°C until RNA extraction. Besides, all participants signed the written informed consent before enrollment, and the protocol of this study was approved by the ethics committee of the Fourth Hospital of Hebei Medical University.

### Cell Culture

Human CRC cell lines (SW837, LS1034, LS513, and HCT116) and human colorectal epithelial cell line CCD-841CoN (ATCC, Manassas, Va, USA) were cultured in Dulbecco’s modified Eagle’s medium/Ham’s Nutrient Mixture F12 (Gibco, Life Technologies, Carlsbad, Calif, USA) containing 10% (v/v) fetal bovine serum (Gibco, Life Technologies) and 1% (v/v) Penicillin Streptomycin-Glutamine (100×, Gibco, Life Technologies) at 37°C with 5% CO_2_.

### Cell Transfection

miR-433-3p mimic, miR-433-3p inhibitor, siRNA sequence targeting lncRNA IGFL2-AS1, PAK4 overexpression plasmid pcDNA3.1-PAK4 (oe-PAK4), pcDNA3.1-IGFL2-AS1 (oe-IGFL2-AS1), and their corresponding controls (empty plasmid was used for overexpression control) were all provided by GenePharma (Shanghai, China) and transfected into cells following the instructions of Lipofectamine 2000 (Invitrogen, Carlsbad, Calif, USA). The transfection efficiency was detected 24 hours after transfection.

### Detection of Relative Gene Expression by the Quantitative Real-Time Polymerase Chain Reaction Method

The total RNA was extracted from cells and tissues using the PureLink RNA microextraction kit (12193016, Thermo Fisher, Waltham, Mass, USA). RNA and miRNA were reverse-transcribed into the complementary DNA using the RevertAid RT reverse transcription kit (K1691, Thermo Fisher) and TaqMan™ MicroRNA Reverse Transcription Kit (4366597, Thermo Fisher). Quantitative real-time polymerase chain reaction (qRT-PCR) was conducted using SYBR® Premix Ex Taq^TM^ II (Perfect Real Time) kit (DRR081, Takara, Tokyo, Japan) and RT qPCR instrument (ABI 7500, ABI, Foster City, Calif, USA) and amplified with 2 steps. The first step was pre-denaturation at 95°C for 30 seconds, and the second step was 40 thermal cycles of 95°C for 5 seconds and 60°C for 34 seconds. Each sample was provided with 3 duplicate wells. The synthesis of PCR primers was carried out by Sangon Biotech (Shanghai, China) (Primer sequences are shown in Table 1). The cycle threshold (Ct) value of each well was recorded. The relative gene expression was calculated based on the 2^−ΔΔCt^ method, with glyceraldehyde-3-phosphate dehydrogenase or U6 as the internal control. ΔΔCt = (mean Ct value of target genes from the experiment group − mean Ct value of housekeeping genes from the experiment group) − (mean Ct value of target genes from the control group − mean Ct value from housekeeping genes of the control group).^20^

### 3-(4,5-Dimethylthiazol-2-yl)-2,5-diphenyltetrazolium Bromide Assay

Upon transfection, cells (5 × 10^4^ cells/mL) were cultured in 96-well plates for 0 hour, 24 hours, 48 hours, and 72 hours, respectively. Then, each well was supplemented with 5 mg/mL 3-(4,5-dimethylthiazol-2-yl)-2,5-diphenyltetrazolium bromide (MTT) solution (20 μL, Sigma, St Louis, Mo, USA). After 4 hours, the MTT solution was replaced by dimethylsulfoxide. The absorbance at a wavelength of 490 nm was determined using a microplate reader.

### Colony Formation Assay

Upon transfection, cells (200 cells/well) were cultured in 6-well plates for 2 weeks. After cell culture, the culture medium was discarded and cells were subjected to paraformaldehyde (4%, Sigma)-fixation at room temperature for 20 minutes. After washing with phosphate buffer saline, cells were stained with 0.1% crystal violet solution (Sigma) for 30 minutes. The colonies were counted through observation using an inverted microscope (100-folds, Olympus, Tokyo, Japan), and the data were processed by Image J version 1.52 software.

### Bioinformatics Analysis

The expressions of lncRNA IGFL2-AS1 and miR-433-3p in CRC and their binding sites with downstream targets were predicted with the help of Starbase (https://starbase.sysu.edu.cn/)^21^ and TargetScan (http://www.targetscan.org/vert_80/)^22^ databases. The subcellular localization of lncRNA IGFL2-AS1 was predicted with the help of the lncLocator database (http://www.csbio.sjtu.edu.cn/bioinf/lncLocator/?tdsourcetag=s_pcqq_aiomsg).^23^

### Nuclear/Cytosol Fractionation Assay

After centrifugation, the supernatant was discarded and precipitates were kept. Then, precipitates were resuspended with hypotonic buffer A [protease inhibitor, RNase inhibitor (N8080119, Thermo Fisher Scientific), 10 mM 4-(2-hydroxyethyl)-1-iperazineethanesulfonic acid (HEPES) (pH7.5), 0.5 mM dithiothreitol (DTT), 10 mM KCl, 1.5 mM MgCl_2_]. Upon complete dispersion, cells were incubated on ice for 10 minutes and centrifuged using a low-speed freezing centrifuge (1000 × g, 4°C) for 10 minutes. Next, the supernatant was taken and placed at a high-speed centrifuge (15 000 × g) for 15 minutes to obtain the cytoplasm. Subsequently, the supernatant was discarded and precipitates were washed with hypotonic buffer twice and resuspended with hypotomic buffer B [10 mM HEPES (pH 7.5), 10 mM KCl, 1.5 mM MgCl_2_, 0.5 mM DTT, 0.5% Nonidet P-40]. After 30 minutes of incubation at 4°C and 10 minutes of centrifugation using a low-speed centrifuge (6000 × g, 4°C), the supernatant was discarded and precipitates were washed with hypotonic buffer once and resuspended with radio immunoprecipitation assay (RIPA) buffer [protease inhibitor, RNase inhibitor, 50 mM Tris HCl (pH 7.5), 1500 mM KCl, 1% Nonidet P-40, 0.5% sodium deoxycholate, 0.1% SDS, 1 mM ethylene diamine tetraacetic acid (EDTA) (pH 8.0)]. After 30 minutes of incubation at 4°C and 20 minutes of centrifugation using a high-speed centrifuge (15 000 × g), the supernatant was collected as cell nuclei.

### Dual-Luciferase Reporter Gene Assay

The binding sequences of miR-433-3p in lncRNA IGFL2-AS1 (IGFL2-AS1-WT) and PAK4 3’UTR (PAK4-WT) and their mutant sequences (IGFL2-AS1-MUT and PAK4-MUT) were cloned into pMIR-Reporter plasmids (Beijing Huayueyang Biotechnology, Beijing, China). The above constructed vectors, miR-433-3p mimic, and mimic NC were transfected into cells. After 48 hours, cells were collected and lysed, and the luciferase activity was detected using the luciferase reporter gene kit (LUC1, Sigma).

### RNA Immunoprecipitation Assay

RNA immunoprecipitation (RIP) assay was performed following the instructions of the Imprint RNA immunoprecipitation kit (Sigma). Briefly, the cell lysates were immunoprecipitated with anti-AGO2 (Ab186733, Abcam, Cambridge, Mass, USA) and anti-IgG (Sigma). LncRNA IGFL2-AS1 and miR-433-3p levels in precipitates were determined using qRT-PCR. The experiment of each group was performed 3 times independently.

### Statistical Analysis

Data analysis and graphing were processed with the help of SPSS 21.0 statistical software (IBM Corp, Armonk, NY, USA) and GraphPad Prism 8.0 software (GraphPad Software Inc., San Diego, Calif, USA). All data were represented as mean ± standard deviation and confirmed to normal distribution and homogeneity of variance. The *t*-test was adopted for pairwise comparisons, 1-way or 2-way analysis of variance (ANOVA) was adopted for multi-group comparisons, and Tukey’s multiple comparison test was adopted for post-test. Pearson’s correlation coefficient analysis was used for correlation analysis. *P-*value was obtained from the 2-sided test and a value of *P*  < .05 meant statistically significant.

## Results

### Lncrna IGFL2-AS1 Was Abundantly Expressed in Colorectal Cancer Tissues and Cells

**Figure 1.**
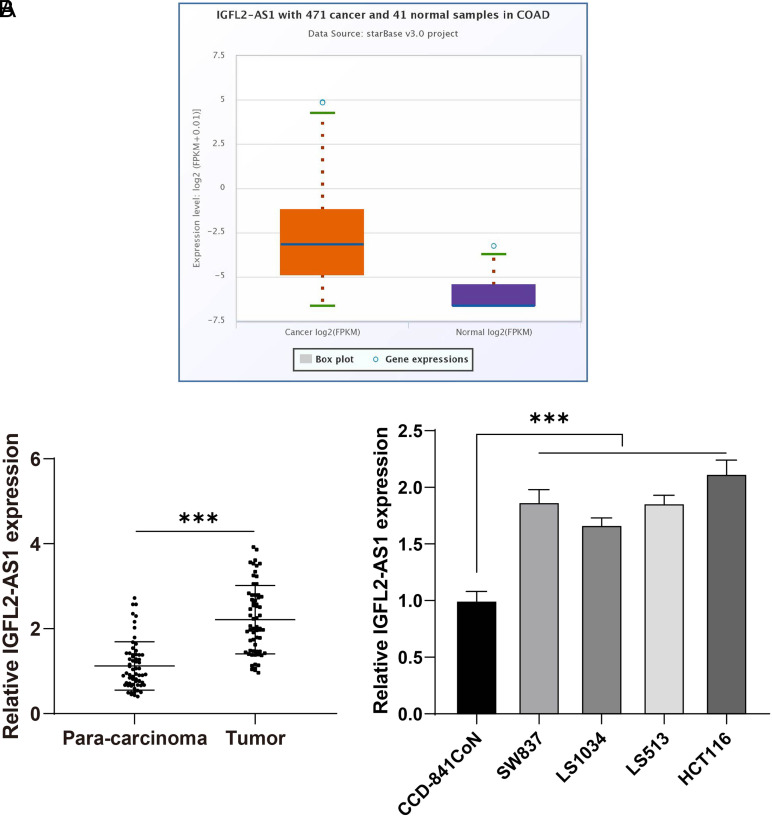
LncRNA IGFL2-AS1 was highly expressed in CRC tissues and cells. (A) LncRNA IGFL2-AS1 expression was predicted by the Starbase database; (B) IGFL2-AS1 expression in tissues (n = 63) and cell lines (CCD-841CoN, SW837, LS1034, LS513, and HCT116) was detected via qRT-PCR. Cell experiments were performed 3 times independently. Data were represented as mean ± SD, *** P < .001. Data in figure B were analyzed using paired *t*-test (left) and 1-way ANOVA (right), followed by Tukey’s multiple comparison test. SD, standard deviation; ANOVA, analysis of variance; lncRNA IGFL2-AS1, long noncoding RNA insulin-like growth factor 2-like family member 2 antisense RNA 1; qRT-PCR, quantitative real-time polymerase chain reaction.

To analyze the expression pattern of lncRNA IGFL2-AS1 in CRC, first, the Starbase database was used to predict lncRNA IGFL2-AS1 expression, which showed that lncRNA IGFL2-AS1 was elevated in CRC (Figure 1A). Then, CRC tissues, CRC cell lines (SW837, LS1034, LS513, and HCT116), and colonic epithelial cell line (CCD-841CoN) were collected for detection of lncRNA IGFL2-AS1. It was found that lncRNA IGFL2-AS1 expression in CRC tissues and cells was higher than that in para-cancerous tissues and CCD-841CoN cells (*P*  < .05, Figure 1B). The above results elucidated that lncRNA IGFL2-AS1 was abundantly expressed in CRC tissues and cells.

### Silencing Lncrna IGFL2-AS1 Discouraged HCT116 Cell Malignant Proliferation

**Figure 2.**
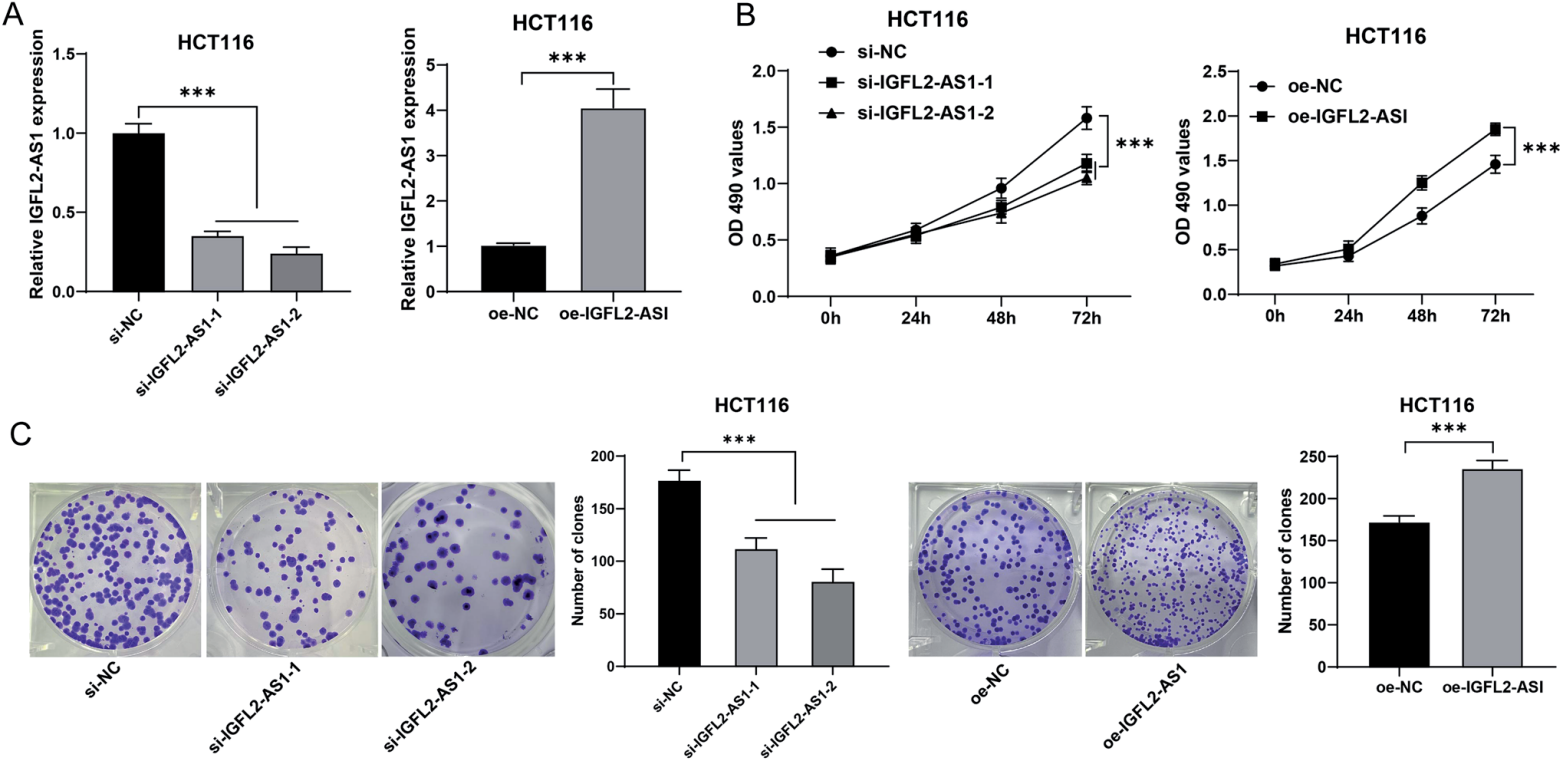
LncRNA IGFL2-AS1 regulated HCT116 cell malignant proliferation. HCT116 cells were transfected with si-IGFL2-AS1-1 and si-IGFL2-AS1-2, with si-NC as the control, and transfected with pcDNA3.1-IGFL2-AS1 (oe-IGFL2-AS1), with empty vector (oe-NC) as the control. (A) Transfection efficiency of si-IGFL2-AS1-1, si-IGFL2-AS1-2, and oe-IGFL2-AS1 was determined via qRT-PCR; (B) Cell viability was assessed via MTT assay; (C) Cell proliferation was assessed via colony formation assay. Cell experiments were performed 3 times independently. Data were represented as mean ± SD, ***P < .001. The multiple-comparisons in figures A and C were analyzed using 1-way ANOVA, and pairwise comparisons were analyzed using the *t* test. Data in figure B were analyzed using 2-way ANOVA, followed by Tukey’s multiple comparison test. SD, standard deviation; ANOVA, analysis of variance; lncRNA IGFL2-AS1, long noncoding RNA insulin-like growth factor 2-like family member 2 antisense RNA 1; qRT-PCR, quantitative real-time polymerase chain reaction; MTT, 3-[4,5-dimethylthiazol-2-yl]-2,5 diphenyl tetrazolium bromide.

To further analyze the regulatory mechanism of lncRNA IGFL2-AS1 in CRC, HCT116 cells with comparatively higher expression of lncRNA IGFL2-AS1 were selected for the subsequent experiments. HCT116 cells were transfected with 2 strands of siRNA to downregulate lncRNA IGFL2-AS1 and transfected with IGFL2-AS1 overexpression vector to upregulate lncRNA IGFL2-AS1 (*P*  < .05, Figure 2A). Next, the MTT assay showed that compared with the si-NC group, cell viability was declined in the si-IGFL2-AS1-1 and si-IGFL2-AS1-2 groups; compared with the oe-NC group, cell viability was enhanced in the oe-IGFL2-AS1 group (*P*  < .05, Figure 2B). Colony formation assay showed that compared with the si-NC group, the clone number was decreased in the si-IGFL2-AS1-1 and si-IGFL2-AS1-2 groups; compared with the oe-NC group, the clone number was increased in the oe-IGFL2-AS1 group (*P*  < .05, Figure 2C). The above results elucidated that silencing lncRNA IGFL2-AS1 discouraged HCT116 cell malignant proliferation and upregulating lncRNA IGFL2-AS1 facilitated HCT116 cell malignant proliferation.

### Lncrna IGFL2-AS1 Bound to mir-433-3p and Inhibited mir-433-3P Expression

**Figure 3.**
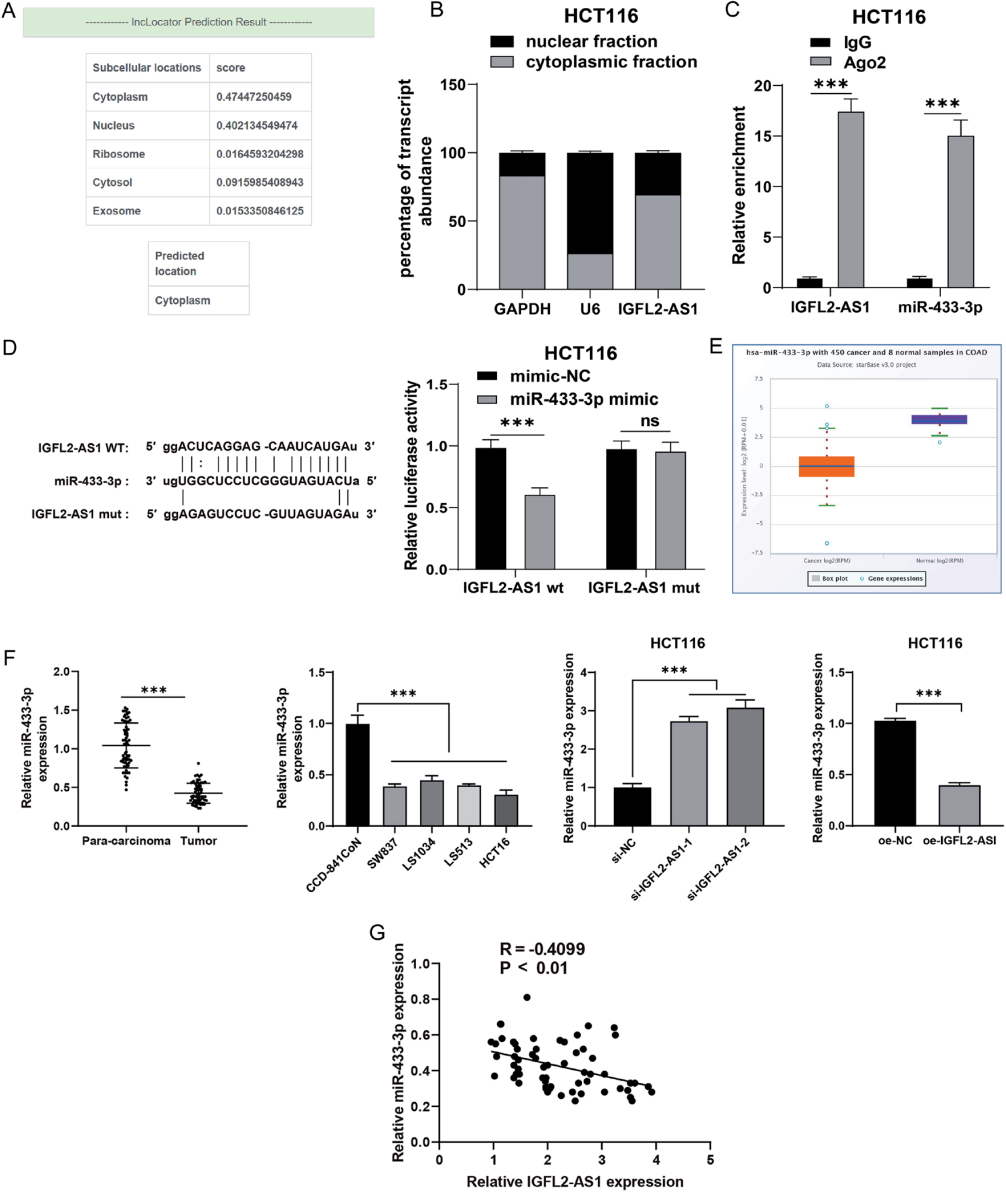
LncRNA IGFL2-AS1 bound to miR-433-3p and inhibited miR-433-3P expression. (A) The subcellular localization of lncRNA IGFL2-AS1 was predicted via the lncLocator database. (B) LncRNA IGFL2-AS1 expression was detected via nuclear/cytosol fractionation assay, with GAPDH as the internal reference of cytoplasm and U6 as the internal reference of nucleus. (C-D) The binding relationship between lncRNA IGFL2-AS1 and miR-433-3p was testified via RIP (C) and dual-luciferase reporter gene assays (D). (E) miR-433-3p expression in CRC was predicted by the Starbase database. (F) miR-433-3p expression in tissues (n = 63) and cells were detected via qRT-PCR. (G) Correlation analysis of lncRNA IGFL2-AS1 and miR-433-3p. Data were represented as mean ± SD, ***P < .001, ns: *P*  > .05. Data in figures C and D were analyzed using 2-way ANOVA, and data in figure F were analyzed using paired *t*-test (left and right) and 1-way ANOVA (middle), followed by Tukey’s multiple comparison test. Data in figure G were analyzed using Pearson’s correlation coefficient. SD, standard deviation; ANOVA, analysis of variance; lncRNA IGFL2-AS1, long noncoding RNA insulin-like growth factor 2-like family member 2 antisense RNA 1; qRT-PCR, quantitative real-time polymerase chain reaction; GAPDH, glyceraldehyde-3-phosphate dehydrogenase; RIP, RNA immunoprecipitation.

To analyze the downstream mechanism of lncRNA IGFL2-AS1 in CRC, lncLocator was adopted to predict the subcellular localization of lncRNA IGFL2-AS1, which showed the location of lncRNA IGFL2-AS1 in the cytoplasm (Figure 3A). Nuclear/cytosol fractionation assay further testified that lncRNA IGFL2-AS1 was mainly expressed in the cytoplasm of HCT116 cells (Figure 3B), indicating that lncRNA IGFL2-AS1 could act as a ceRNA to realize its effects in CRC cell malignant proliferation. Therefore, we screened downstream target genes of lncRNA IGFL2-AS1 through the Starbase database, and attention was paid to miR-433-3p. Prior research demonstrated that miR-433 is weakly expressed in CRC, and miR-433 overexpression could inhibit CRC cell malignant proliferation.^15^ The binding relationship between lncRNA IGFL2-AS1 and miR-433-3p was further testified via the RIP and dual-luciferase reporter gene assays (*P*  < .05, Figure 3C-D). Besides, the Starbase database predicted that miR-433-3p was weakly expressed in CRC (*P*  < .05, Figure 3E). Moreover, qRT-PCR showed that miR-433-3p was weakly expressed in CRC tissues and cells, miR-433-3p was both markedly increased in the si-IGFL2-AS1-1 and si-IGFL2-AS1-2 groups compared with the si-NC group and markedly declined in the oe-IGFL2-AS1 group compared with the oe-NC group (*P*  < .05, Figure 3F), and miR-433-3p was negatively correlated with lncRNA IGFL2-AS1 in CRC tissues (*P*  < .05, Figure 3G). The above results elucidated that lncRNA IGFL2-AS1 bound to miR-433-3p and inhibited miR-433-3P expression.

### mir-433-3p Downregulation Partly Reversed the Inhibition of Silencing Lncrna IGFL2-AS1 on HCT116 Cell Malignant Proliferation

**Figure 4.**
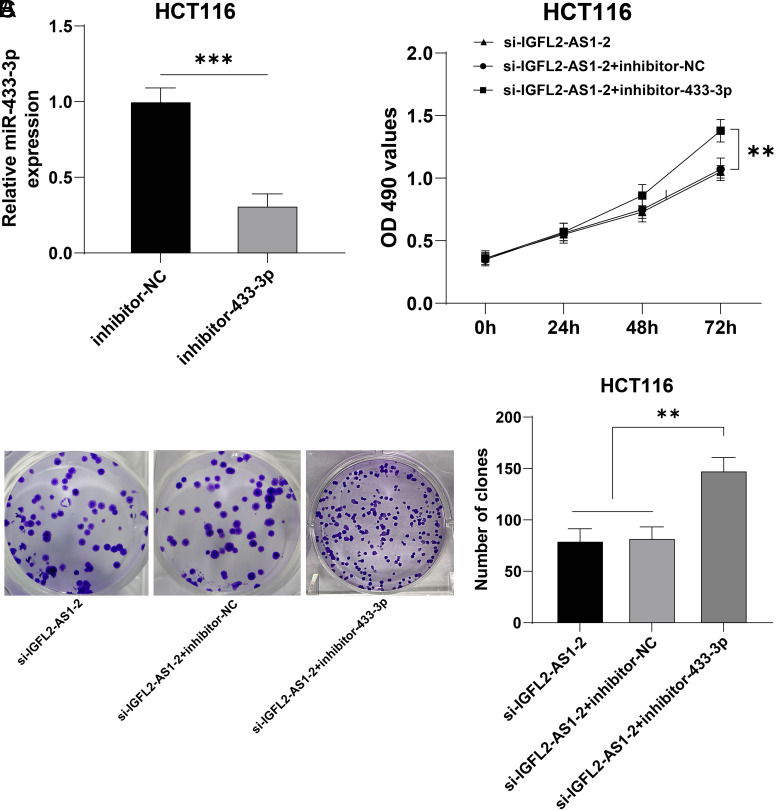
miR-433-3p downregulation partly reversed the inhibition of silencing lncRNA IGFL2-AS1 on HCT116 cell malignant proliferation. HCT116 cells were transfected with inhibitor-433-3p, with inhibitor-NC as the control. (A) The transfection efficiency of inhibitor-433-3p was detected via qRT-PCR, followed by a collaborative experiment with si-IGFL2-AS1-2. (B) Cell viability was assessed via MTT assay. (C) Cell proliferation was assessed via colony formation assay. Cell experiments were performed 3 times independently. Data were represented as mean ± SD, ***P < .001, **P < .01. Data in figure A were analyzed using unpaired *t*-test, data in figure B were analyzed using 2-way ANOVA, and data in figure C were analyzed using 1-way ANOVA, followed by Tukey’s multiple comparison test. SD, standard deviation; ANOVA, analysis of variance; lncRNA IGFL2-AS1, long noncoding RNA insulin-like growth factor 2-like family member 2 antisense RNA 1; qRT-PCR, quantitative real-time polymerase chain reaction; MTT, 3-[4,5-dimethylthiazol-2-yl]-2,5 diphenyl tetrazolium bromide.

Next, a functional rescue experiment was designed to validate the above mechanism. HCT116 cells were transfected with inhibitor-433-3p, with inhibitor-NC as the control. Quantitative real-time polymerase chain reaction showed that compared with the inhibitor-NC group, miR-433-3p was markedly diminished in the inhibitor-433-3p group (*P*  < .05, Figure 4A). Then, inhibitor-433-3p was used to perform a collaborative experiment with si-IGFL2-AS1-2 which had a higher interference efficiency. Our results showed that compared with the si-IGFL2-AS1-2 + inhibitor-NC group, cell viability and clone number were augmented in the si-IGFL2-AS1-2 + inhibitor-433-3p (*P*  < .05, Figure 4B and C). The above results elucidated that miR-433-3p downregulation partly reversed the inhibition of silencing lncRNA IGFL2-AS1 on HCT116 cell malignant proliferation.

### mir-433-3p Inhibited Pak4 Transcription

**Figure 5.**
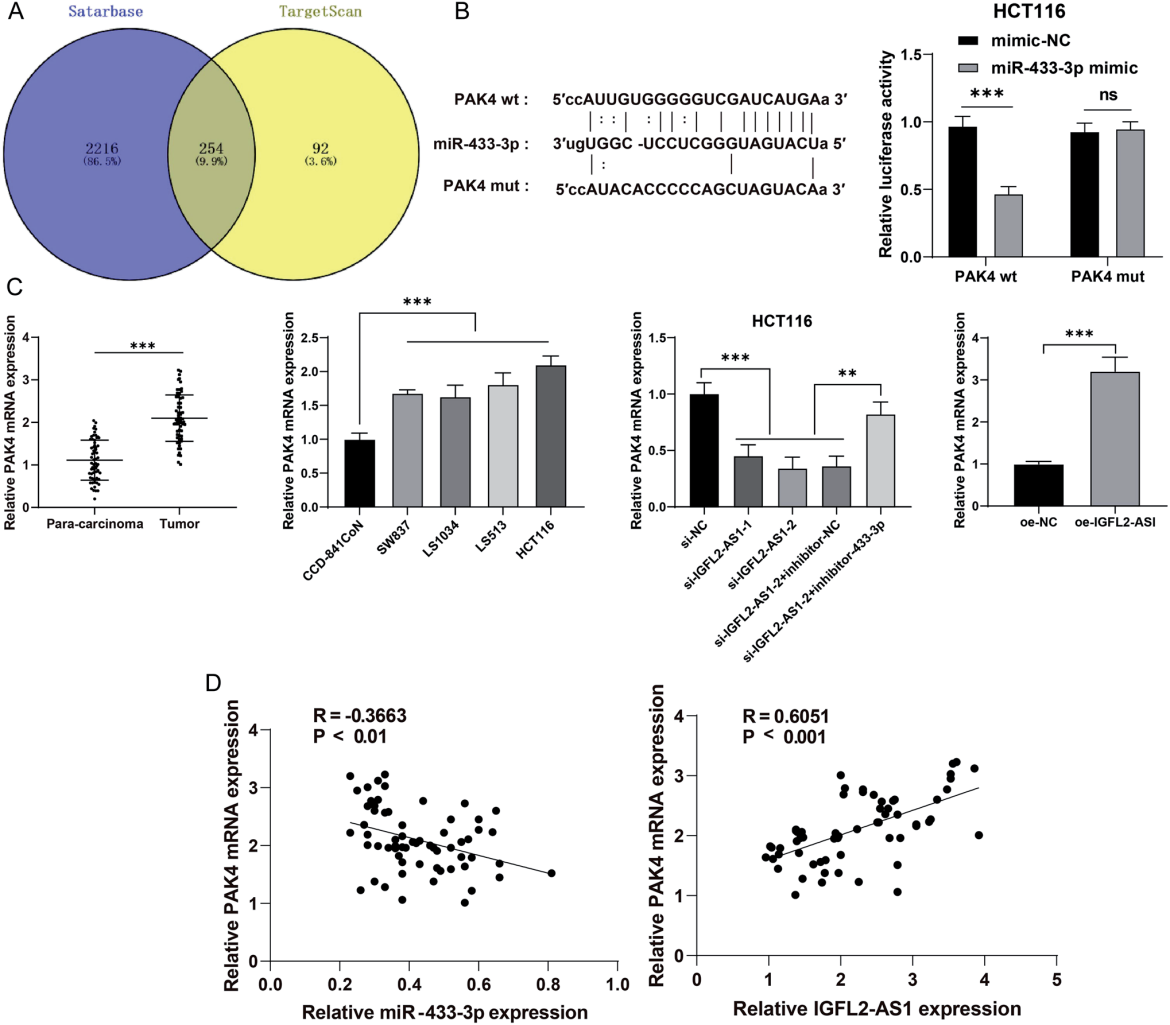
miR-433-3p inhibited PAK4 transcription. (A) Downstream target genes of miR-433-3p was screened by Starbase and TargetScan databases, and intersections were identified. (B) The binding relationship between PAK4 and miR-433-3p was testified via the dual-luciferase reporter gene assay. (C) mRNA level of PAK4 in CRC tissues (n = 63) (left) and cell lines (CCD-841CoN, SW837, LS1034, LS513, and HCT116) (middle) and all experimental groups (right) was determined via qRT-PCR. (D) Correlation analysis of miR-433-3p and PAK4 mRNA, lncRNA IGFL2-AS1 and PAK4 mRNA. Cell experiments were performed 3 times independently. Data were represented as mean ± SD, ns: *P*  > .05, ***P < .001, **P < .01. Data in figure B were analyzed using 2-way ANOVA, data in figure C were analyzed using paired *t*-test (left and right) and 1-way ANOVA (middle), followed by Tukey’s multiple comparison test. Data in figure D were analyzed using Pearson’s correlation coefficient. SD, standard deviation; ANOVA, analysis of variance; lncRNA IGFL2-AS1, long noncoding RNA insulin-like growth factor 2-like family member 2 antisense RNA 1; PAK4, p21 (RAC1)-activated kinase 4.

To further explore the regulatory mechanism of miR-433-3p, downstream target genes of miR-433-3p were predicted through the Starbase database, and intersections were identified (Figure 5A), in which PAK4 was selected as a target gene of miR-433-3p. Prior research has highlighted that PAK4 could repress cancer cell proliferation and migration.^24^ The binding relationship between PAK4 and miR-433-3p was testified via the dual-luciferase reporter gene assay (*P*  < .05, Figure 5B). Quantitative real-time polymerase chain reaction showed that the mRNA level of PAK4 was increased in CRC tissues and cells compared with para-cancerous tissues and CCD-841CoN cells, while decreased in the si-IGFL2-AS1-1 and si-IGFL2-AS1-2 groups compared with the si-NC group, elevated in the si-IGFL2-AS1-2 + inhibitor-433-3p group compared with the si-IGFL2-AS1-2 + inhibitor-NC, and increased in the oe-IGFL2-AS1 group compared with the oe-NC group (*P*  < .05, Figure 5C). PAK4 mRNA was negatively correlated with miR-433-3p and positively correlated with lncRNA IGFL2-AS1 in CRC tissues (Figure 5D). The above results elucidated that miR-433-3p inhibited PAK4 transcription.

### PAK4 Overexpression Partly Reversed the Inhibition of Silencing Lncrna IGFL2-AS1 on HCT116 Cell Malignant Proliferation

**Figure 6.**
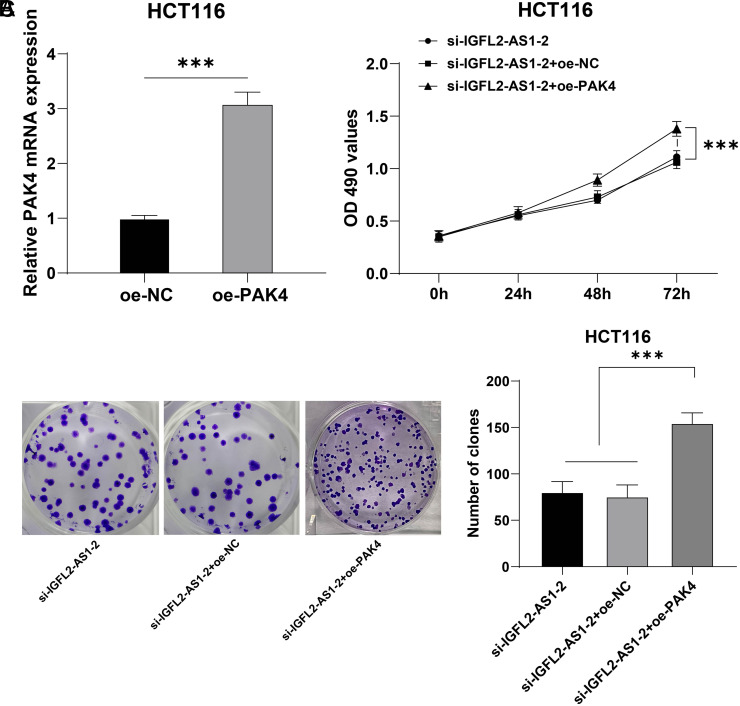
PAK4 overexpression partly reversed inhibition of silencing lncRNA IGFL2-AS1 on HCT116 cell malignant proliferation. HCT116 cells were transfected with the overexpression vector pcDNA3.1-PAK4 (oe-PAK4), with the empty vector pcDNA-3.1 (oe-NC) as the control. (A) The transfection efficiency of oe-PAK4 was detected via qRT-PCR, followed by a collaborative experiment with si-IGFL2-AS1-2. (B) Cell viability was assessed via MTT assay. (C) Cell proliferation was assessed via colony formation assay. Cell experiments were performed 3 times independently. Data were represented as mean ± SD, ***P < .001. Data in figure A were analyzed using unpaired *t*-test, data in figure B were analyzed using 2-way ANOVA, and data in figure C were analyzed using 1-way ANOVA, followed by Tukey’s multiple comparison test. SD, standard deviation; ANOVA, analysis of variance; lncRNA IGFL2-AS1, long noncoding RNA insulin-like growth factor 2-like family member 2 antisense RNA 1; qRT-PCR, quantitative real-time polymerase chain reaction; MTT, 3-[4,5-dimethylthiazol-2-yl]-2,5 diphenyl tetrazolium bromide; PAK4, p21 (RAC1)-activated kinase 4.

Another functional rescue experiment was performed to validate the above regulatory mechanism. First, HCT116 cells were transfected with oe-PAK4 to upregulate the mRNA level of PAK4 (*P*  < .05, Figure 6A). Then, oe-PAK4 was used to perform a collaborative experiment with si-IGFL2-AS1-2. Our results showed that compared with the si-IGFL2-AS1-2 and si-IGFL2-AS1-2 + oe-NC groups, cell viability and clone number were markedly increased in the si-IGFL2-AS1-2 + oe-PAK4 group (*P*  < .05, Figure 6B and C). The above results elucidated that PAK4 overexpression partly reversed the inhibition of silencing lncRNA IGFL2-AS1 on HCT116 cell malignant proliferation.

## Discussion

Genetic alterations participate in cancer cell behaviors, such as proliferation, migration, invasion, and apoptosis.^25^ Exploring the ceRNA network of lncRNA/miRNA/mRNA helps to better understand the molecular etiology of CRC and provides therapeutic targets in clinic management.^26,27^ In the current study, our findings unveiled that lncRNA IGFL2-AS1 comparatively binds to miR-433-3p to facilitate PAK4 transcription, thus promoting HCT116 cell malignant proliferation.

Long noncoding RNAs are crucial players in CRC tumorigenesis and metastasis and are potential indicators of patients’ prognosis.^28^ Long noncoding RNA IGFL2-AS1 exerts oncogenic functions in various cancers, and its upregulation predicts a poor prognosis.^7-9^ However, its expression pattern and role in CRC remain elusive. In our study, the Starbase database predicted that lncRNA IGFL2-AS1 was abundantly expressed in CRC, and increased lncRNA IGFL2-AS1 was found in CRC tissues and cells (SW837, LS1034, LS513, and HCT116). To further evaluate the significance of lncRNA IGFL2-AS1 on CRC, lncRNA IGFL2-AS1 was downregulated in HCT116 cells via siRNA, and we observed that lncRNA IGFL2-AS1 downregulation reduced cell viability and clone number. In accordance, a former study by Cen et al^7^ reported that lncRNA IGFL2-AS1 facilitates CRC cell proliferation, migration, and invasion and high lncRNA IGFL2-AS1 level predicts a worse prognosis of colon cancer. Our study highlights the novelty in its focus on CRC malignant proliferation and exploration of its profound downstream mechanism. Moreover, lncRNA IGFL2-AS1 also plays an oncogenic role in tongue squamous cell carcinoma and basal-like breast cancer.^9,29^ Moreover, increased lncRNA IGFL2-AS1 is associated with the tumor-node-metastasis staging of colon cancer and renal cell carcinoma.^7,30^ Altogether, our findings and shreds of evidence suggested that lncRNA IGFL2-AS1 is upregulated in CRC tissues and cells and plays a driving role in HCT116 cell malignant proliferation.

Prior studies have evidenced that lncRNA IGFL2-AS1 could function as a ceRNA in gastric cancer.^8,10^ Through the lncLocator database and nuclear/cytosol fractionation assay, we validated that lncRNA IGFL2-AS1 was mainly expressed in the cytoplasm of HCT116 cells, suggesting that lncRNA IGFL2-AS1 could play a role as a ceRNA in CRC. miRNA, as a critical player in the ceRNA regulatory network, regulates CRC progression, metastasis, and therapeutic resistance and links with risk factors related to oncogenesis in CRC, such as obesity and insulin resistance.^31-33^ Of note, miR-433 was previously found to be decreased in CRC and suppress CRC cell motility.^14,15,34^ We confirmed the binding relationship between lncRNA IGFL2-AS1 and miR-433-3p via the database prediction, RIP, and dual-luciferase reporter gene assays. Decreased miR-433-3p was found in CRC tissues and cells, and miR-433-3p was negatively correlated with lncRNA IGFL2-AS1 in CRC tissues. To explore the role of miR-433-3p in CRC, miR-433-3p was downregulated in HCT116 cells in the si-IGFL2-AS1 group via inhibitor-433-3p, and we observed that miR-433-3p downregulation elevated cell viability and clone number. Therefore, our findings identified the role of miR-433-3p downregulation in promoting CRC cell through negative correlation with lncRNA IGFL2-AS1, which is supported by another study by Hong et al^11^ demonstrating that miR-433-3p overexpression restrains CRC cell proliferation, invasion, and epithelial–mesenchymal transition through negative correlation with lncRNA LINC00460. Consistently, miR-433-3p also serves as a tumor suppressor in non-small cell lung cancer, bladder cancer, osteosarcoma, and breast cancer.^12,13,35,36^

Thereafter, we focused on the downstream mechanism of miR-433-3p. Prior research has shed light on a binding relationship between miR-433-3p and PAK4 in oral squamous cell carcinoma.^16^ Besides, their binding relationship in CRC was further testified via database prediction and dual-luciferase reporter gene assay. PAK4 was found to be abundantly expressed in CRC tissues and cells and showed a negative correlation with miR-433-3p and a positive correlation with lncRNA IGFL2-AS1. PAK4, an oncoprotein that overcomes oncogene-induced senescence, shows upregulation in CRC and encourages CRC cell proliferation, migration, and invasion and tumor growth.^17-19,37^ To investigate the role of PAK4 in CRC, PAK4 was overexpressed in HCT116 cells in the si-IGFL2-AS1 group via oe-PAK4, and we observed that PAK4 overexpression augmented cell viability and clone number. In line with these, the oncogenic property of PAK4 was also manifested in breast cancer, bladder cancer, pancreatic cancer, and lung cancer.^38-41^ Above all, PAK4 overexpression partly reversed the inhibition of silencing lncRNA IGFL2-AS1 on HCT116 cell malignant proliferation. Yet, the interactions between lncRNA IGFL2-AS1 and miR-433-3p and PAK4 in CRC have not been reported before, which further underpins the novelty of our study.

To conclude, this study is the first of its kind to disclose the ceRNA network of lncRNA IGFL2-AS1/miR-433-3p/PAK4 in CRC, which provides a novel targeted therapeutic target for CRC. However, this study failed to perform the power calculation to evaluate the sample size, or investigate other downstream targets of lncRNA IGFL2-AS1 in CRC, except miR-433-3p and PAK4, or testify to the role of lncRNA IGFL2-AS1/miR-433-3p/PAK4 in animals. In the next step, the role of lncRNA IGFL2-AS1 in CRC will be further explored with the analysis of downstream mechanisms and the establishment of animal models.

## Figures and Tables

**Table 1.** qPCR Primers

Gene	Forward Primer (5’-3’)	Reverse Primer (5’-3’)
IGFL2-AS1	TCATATGGACTCATGTGACATTTGG	AGGTCATGTGGGTCATGTATCCACTG
miR-433-3p	GCCGAGATCATGATGGGCTCCT	CTCAACTGGTGTCGTGGA
PAK4	GAGAATTCTCTGCTGCAGCCATGTTCAGCA	CTCTCGAGTCATCTCATGCGGTTTTGTCTC
U6	CTCGCTTCGGCAGCACA	AACGCTTCACGAATTTGCGT
GAPDH	CAGTCACTACTCAGCTGCCA	GAGGGTGCTCC GGTAG

lncRNA IGFL2-AS1, long noncoding RNA insulin-like growth factor 2-like family member 2 antisense RNA 1; GAPDH, glyceraldehyde-3-phosphate dehydrogenase; PAK4, p21 (RAC1)-activated kinase 4.
